# DJ-1 based peptide, ND-13, promote functional recovery in mouse model of focal ischemic injury

**DOI:** 10.1371/journal.pone.0192954

**Published:** 2018-02-28

**Authors:** Lior Molcho, Tali Ben-Zur, Yael Barhum, Daniel Offen

**Affiliations:** 1 Sagol School of Neuroscience, Tel-Aviv University, Tel Aviv, Israel; 2 Laboratory of Neuroscience, Felsenstein Medical Research Center, Sackler Faculty of Medicine, Tel Aviv University, Tel Aviv, Israel; University of South Florida, UNITED STATES

## Abstract

Stroke is a leading cause of death worldwide and inflicts serious long-term damage and disability. The vasoconstrictor Endothelin-1, presenting long-term neurological deficits associated with excitotoxicity and oxidative stress is being increasingly used to induce focal ischemic injury as a model of stroke. A DJ-1 based peptide named ND-13 was shown to protect against glutamate toxicity, neurotoxic insults and oxidative stress in various animal models. Here we focus on the benefits of treatment with ND-13 on the functional outcome of focal ischemic injury. Wild type C57BL/6 mice treated with ND-13, after ischemic induction in this model, showed significant improvement in motor function, including improved body balance and motor coordination, and decreased motor asymmetry. We found that DJ-1 knockout mice are more sensitive to Endothelin-1 ischemic insult than wild type mice, contributing thereby additional evidence to the widely reported relevance of DJ-1 in neuroprotection. Furthermore, treatment of DJ-1 knockout mice with ND-13, following Endothelin-1 induced ischemia, resulted in significant improvement in motor functions, suggesting that ND-13 provides compensation for DJ-1 deficits. These preliminary results demonstrate a possible basis for clinical application of the ND-13 peptide to enhance neuroprotection in stroke patients.

## Introduction

Stroke is the second most common cause of death, causing 9% of all deaths around the world, and is the most frequent cause of permanent disability in adults worldwide [1]. When the flow of blood to the brain is suddenly stopped, neuronal function is impaired and pathological pathways are triggered, causing irreversible neuronal damage in the ischemic area within minutes of onset. In the hours and days following stroke, the damaged regions undergo a broad scale necrosis, which causes the death of many types of cells [1–3]. Failure of energy production causes a flood of neurotransmitters to be released from neurons, mostly release of the excitatory glutamate, which further amplifies the damage [4,5]. Subsequent processes, such as oxidative stress, dysfunction of blood brain barrier and inflammatory response all contribute to the outcome of stroke [6,7].

The high rate of oxidative metabolism in the brain renders it vulnerable to oxidative stress. Since the endogenous scavenging mechanisms are normally not high enough to match excess radical formation, reactive oxygen species (ROS) levels increase after ischemic injury, resulting in massive oxidative stress [7,8]. The mitochondria is implicated in this process due to excessive superoxide production during electron transport chain [9,10]. Free radicals are also generated through multiple injury mechanisms such as mitochondrial inhibition, Ca2+ overload, reperfusion injury, and inflammatory response after ischemia [11–13]. Oxidative stress directly damages proteins, lipids, carbohydrates and nucleic-acids, leading to cell dysfunction and DNA fragmentation, contributing to ischemic cell death [8,14]. Oxidative stress also leads to mitochondrial dysfunction. Mitophagy is one of the major mechanisms of mitochondrial quality control, and is mediated by pink1 and Parkin (PARK2) proteins.

Upon mitochondrial depolarization, likely the result of oxidative damage, pink1 is localized to the outer mitochondrial membrane where it recruits, phosphorylates and activates parkin. This causes the ubiquitination of mitochondrial substrates which are degraded by the proteasome, resulting in fragmentation of mitochondria and removal via mitophagy [15–18].

DJ-1, also known as PARK7, has diverse function, such as preserving mitochondrial function, regulating kinase pathways and acting as a transcriptional regulator affecting anti-oxidant genes [19–24]. It has been shown that parkin and DJ-1 interact under oxidative stress conditions, causing an increase in the steady-state levels of DJ-1, and a subsequent decrease in oxidative stress [25–27]. Pink1 and DJ-1 are also recruited when the mitochondrial membrane potential has decreased, resulting in an increase in cell viability [21,25,28]. DJ-1 also provides protection against excitotoxicity and ischemic brain injury [29,30].

We have developed a DJ-1 based peptide named ND-13. ND-13 is a 20-amino acid peptide composed of a 13-amino acids sequence from the DJ-1 protein, attached to a TAT-derived 7-amino acid sequence, that serves as a cell penetrating peptide (CPP) [31–33]. We have shown that ND-13 protects cells against oxidative and neurotoxic insults, reduces reactive oxygen species (ROS) accumulation, and activates protective factors that increase cell survival. Specifically, ND-13 attenuated dopaminergic system dysfunction and improved the behavioral outcome in the 6-hydroxydopamine mouse model of Parkinson's disease [34], and attenuated nigrostriatal degeneration in two models of multiple system atrophy [35].

A large number of processes contribute to the outcome of stroke, and therefore treating a single aspect is probably insufficient. Strategies that combine promotion of tissue integrity and neuroprotection are therefore being increasingly explored as therapeutic targets.

In this study we used the Endothelin-1 (ET-1) focal ischemia model to study the possible neuroprotective effect of ND-13. ET-1 is a potent vasoconstrictor which is produced endogenously during ischemic stroke and which contributes to overall loss of cells and disability. It is often used as a minimally invasive model of focal stroke to evaluate candidate pro-regenerative therapies. One advantage of this model is that it causes highly reproducible infarcts. Another benefit is that it can be used in elderly rodents with only very low resulting mortality. As generally elderly people are the population subset which has increased cerebrovascular risk factors, the Endothelin-1 model seems an appropriate starting point to evaluate the potential efficacy of ND-13 in ischemic stroke.

The aims of the present study are to examine the efficacy of treatment with ND-13 on the functional outcome and recovery following focal ischemic injury in separate cohorts of DJ-1 knockout (DJ-1 KO) mice and C57BL/6 wild type mice and investigate the relevance of DJ-1 to ischemic stroke. We found that treatment with ND-13 significantly improves the motor function of both wild type and DJ-1 KO mice following induced focal ischemia.

## Materials and methods

### Ethics statement

All experimental procedures were approved by the Tel Aviv University Committee of Animal Use for Research and Education. All surgery was performed under subcutaneous injections of a mixture of ketamine (100mg/kg) and xylazine (8mg/kg) anesthesia, and all efforts were made to minimize suffering.

### DJ-1 based peptide: ND-13

ND-13 is a 20-amino acids peptide composed of a 13-amino acids sequence from the DJ-1 protein, attached to a TAT-derived 7-amino acids sequence. The sequence of ND-13 is YGRKKRRKGAEEMETVIPVD. ND-13 was synthesized and provided by ChinaPeptides (China).

### Animals

C57BL/6 male mice at the age of 10–12 weeks were purchased from Harlan, Israel. Transgenic DJ-1 knockout mice were purchased from the Jackson Laboratory (Bar Harbor, ME, USA). Animals were placed in a light-controlled environment (12-h light/ dark cycle) and housed in individually ventilated cages (IVC) with free access to food and water. Animals were acclimatized for 1 week prior to experimentation and randomly divided into experimental groups of 13–14 mice each.

### Surgical procedure and treatment

**Fig 1:**
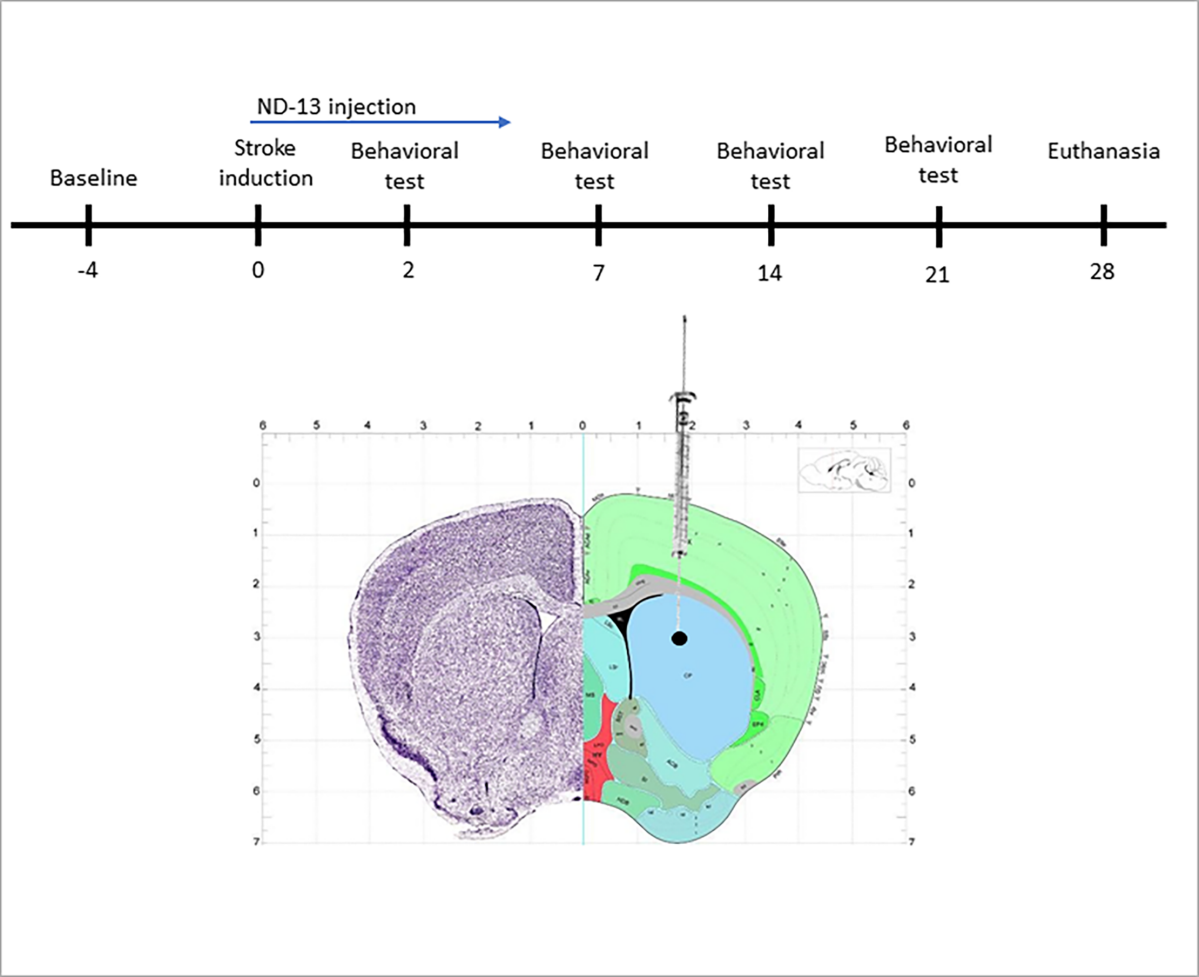
Experimental protocol and ischemic injury location. Experimental protocol in days (A). The vasoconstrictor Endothelin-1 was injected into the striatum to induce ischemia. The ischemic area in the striatum is circled in black (B).

Mice were anesthetized with ketamine and xylazine (100 mg/kg and 8 mg/kg, respectively) and placed in a stereotaxic frame. 5 μl of the vasoconstrictor endothelin-1 (ET-1, 0.2 mg/ml dissolved in sterile saline, Calbiochem, CA, USA) where injected into the right striatum at the following coordinates (relative to bregma): +0.5 mm anterioposterior, +1.9 mm mediolateral, −2.9 mm dorsoventral (infusion rate 0.3 μl/min, Fig 1B). The needle was left in place for 3 additional minutes before withdrawal, and the incision was sutured. Sham operated mice were treated identically except they received injections of sterile saline (instead of ET-1). ND-13 (1mg/ml dissolved in sterile saline) or saline were administered subcutaneously twice a day for five days following surgery, starting 3 hours after ET-1 injection.

### Behavioral tests and analysis

Behavioral tests of were performed after ischemia induction to measure motor function.

**Fig 2:**
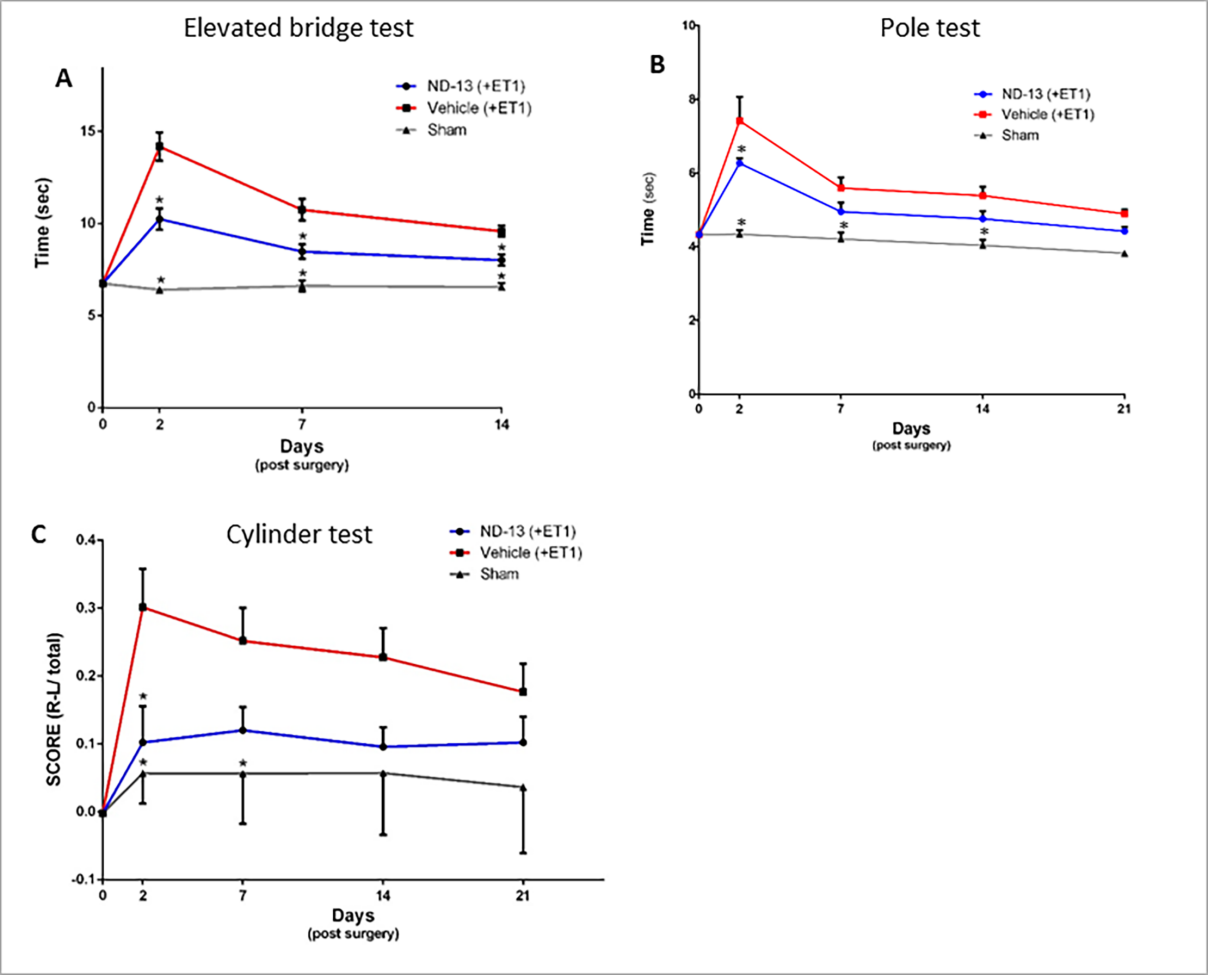
Effect of ND-13 treatment on functional recovery after focal ischemic injury. ND-13 treatment significantly improved time spent crossing the bridge in the elevated bridge test, two days after injury, compared to control (A, p<0.05). The time to descend from a vertical pole in the pole test also decreased following ND-13 treatment compared to control (B, p<0.05). The motor asymmetry in the cylinder test decreased 2 days after ischemic injury (C, p<0.05). ND-13 effect was still consistent 21 days after ischemic injury in all behavioral tests, that is, mice treated with ND-13 perform better than control mice. (Data is shown as mean ± SEM).

The cylinder test, which measures forelimb use during vertical exploration, was performed to as previously described [36]. The final score was calculated as follows: non-impaired forelimb movement − impaired forelimb movement / total (non-impaired + impaired + both forelimb movements). The Cylinder test was performed 2 and 7 days after ischemic injury induction. In the first experiment (presented in Fig 2) a longer analysis of this test was done (21 days) to assess the compatibility of the test to our model and test the effect of repeated testing on the spontaneous activity of the mice.

The Elevated bridge test, which assess motor coordination and balance, was performed as previously described [37]. The score represents the time it took the animal to cross the bridge and get into the goal-box. The elevated bridge test was performed 2, 7 and 14 days after ischemic injury induction.

The Pole test, which assess the locomotor activity, was performed as previously described [38]. The score represents the time it took animals to descend to the floor. The pole test was performed at 2, 7, 14 and 21 days after ischemic injury induction.

### Statistical analysis

Statistical analyses of the data sets were carried out using GraphPad Prism for Windows (Graphpad Software, CA, USA). Statistical significance was determined by two-way ANOVA with repeated measures followed by Dunnett’s post hoc test. Values are presented as mean ± SEM. The results were considered significant at minimal significance level of *p*≤0.05.

### Proteomics analysis

Global quantification of protein expression was done in the De Botton Institute for Protein Profiling (Weizmann institute, Israel). For the proteomic study, two experimental groups of C57BL/6 mice (n = 4 each) were generated as follows: (1) ND-13 treatment following Endothelin-1 injection into the right striatum, (2) Vehicle treatment following Endothelin-1 injection into the right striatum. Treatment was given 3 hours after surgery and twice a day for the next 2 days. Then, mice were sacrificed for striatal protein extraction. Total of 8 mice participated in the experiment in order to decrease intrinsic variability. The protein mix of each individual mouse was analyzed separately and only then averages of the animals in the same groups were calculated. Fold changes of protein expression between the tested groups was also calculated. Changes between groups were considered significant at ±2-fold change and *p*<0.05.

### Sample preparation

Samples (n = 8) were subjected to in-solution tryptic digestion using a modified Filter Aided Sample Preparation protocol (FASP). All chemicals are from Sigma Aldrich (unless stated otherwise). Sodium dodecyl sulfate buffer (SDT) included: 4%(w/v) SDS, 100mM Tris/HCl pH 7.6, 0.1M DTT. Urea buffer A (UA) contained: 8 M urea (Sigma, U5128) in 0.1 M Tris/HCl pH 8.5. Urea buffer B (UB) contained: 8 M urea in 0.1 M Tris/HCl pH 8.0. IAA solution: 0.05 M iodoacetamide in UA. Tissue was homogenized and dissolved in 100μL SDT buffer. Homogenate was centrifuged at 16,000 g for 10min. 100ug total protein were mixed with 200 μL UB and loaded onto 30 kDa molecular weight cutoff filters and centrifuged. 200 μl of UB were added to the filtering unit and centrifuged at 14,000 x g for 40 min. Proteins were alkylated by adding 100 μl IAA and incubating in the dark for 30 min, followed by 2 washes with Ammonium Bicarbonate. Trypsin was then added (50:1 protein amount:trypsin) and samples incubated at 37°C overnight. Additional amount of trypsin was added and incubated for 4 hours at 37°C. Digested proteins were then centrifuged and collected in a clean collecting tube. 50ul Nacl 0.5M was added to the filtering unit and centrifuged. Reaction was stopped by acidifying with 1% trifloroacetic acid. Peptides were desalted using HBL Oasis, Speed vac to dryness and stored in -80°C until analysis.

### Liquid chromatography

ULC/MS grade solvents were used for all chromatographic steps. Each sample was loaded using split-less nanoUltra Performance Liquid Chromatography (10 kpsi nano-Acquity; Waters, Milford, MA, USA). The mobile phase was: A) H2O + 0.1% formic acid and B) acetonitrile + 0.1% formic acid. Dry peptides were dissolved in 97:3 water:acetonitrile (v/v) + 0.1% formic acid solution. Desalting of the samples was performed online using a reversed-phase C18 trapping column (180 μm internal diameter, 20 mm length, 5 μm particle size; Waters). The peptides were then separated using a T3 HSS nano-column (75 μm internal diameter, 250 mm length, 1.8 μm particle size; Waters) at 0.35 μL/min. Peptides were eluted from the column into the mass spectrometer using the following gradient: 4% to 20%B in 140 min, 20% to 90%B in 25 min, maintained at 90% for 5 min and then back to initial conditions.

### Mass spectrometry

The nanoUPLC was coupled online through a nanoESI emitter (10 μm tip; New Objective; Woburn, MA, USA) to a quadrupole orbitrap mass spectrometer (Q Exactive Plus, Thermo Scientific) using a FlexIon nanospray apparatus (Proxeon).

Data was acquired in DDA mode, using a Top20 method. MS1 resolution was set to 70,000 (at 400m/z) and maximum injection time was set to 20 msec. MS2 resolution was set to 17,500 and maximum injection time of 60 msec.

### Data processing and analysis

Raw data was imported into the Expressionist® software (Genedata) and processed as described here [39]. The software was used for retention time alignment and peak detection of precursor peptides. A master peak list was generated from all MS/MS events and sent for database searching using Mascot v2.5 (Matrix Sciences). Data was searched against the mouse protein database from UniprotKB (http://www.uniprot.org/) appended with 125 common laboratory contaminant proteins. Fixed modification was set to carbamidomethylation of cysteines and variable modifications were set to oxidation of methionines and deamidation of N or Q. Search results were then filtered using the PeptideProphet [40] algorithm to achieve maximum false discovery rate of 1% at the protein level. Peptide identifications were imported back to Expressions to annotate identified peaks. Quantification of proteins from the peptide data was performed using an in-house script [39]. Data was normalized base on the total ion current. Protein abundance was obtained by summing the three most intense, unique peptides per protein. A Student’s t-Test, after logarithmic transformation, was used to identify significant differences across the biological replica. Fold changes between treatments were calculated based on the ratio of arithmetic means of the replicate samples.

## Results

### ND-13 improves motor function in a model of focal ischemic injury on wild type mice

The vasoconstrictor ET-1 is being increasingly used to induce focal ischemic injury in rodents as a model of stroke. Animals in this model display significant long-term neurological deficits, associated with excitotoxicity, inflammatory response and oxidative stress [41–44]. In order to study the effects of ND-13 after ischemic injury, 5 μl of ET-1 (0.2 mg/ml) was injected into the right striatum. The location in the striatum in which the insult was administered is circled in black in Fig 1(B). A histological study, using triphenyl tetrazolium chloride (TTC) staining, clearly showed the resulting ischemic damage (data not shown). However, the borders of the damaged area were not sufficiently definable. In view of this fact and the reported lack of correlation between neurological, histological and behavioral outcomes in focal cerebral ischemia rodent studies [45], we decided to determine outcome by measuring behavioral results.

Mice injected with ET-1 showed significant motor deficits, as measured by behavioral tests following ischemic injury compared to sham operated mice. Baseline measurements were taken before the surgery and mice from all C57BL/6 groups showed similar results. Mice received ND-13 or vehicle (saline) twice a day for 5 days after ET-1 injection into the right striatum, starting 3h after ischemic injury infliction. In the elevated bridge test, mice treated with ND-13 were 30% faster compared to the group treated with saline, 2 days after injury (ND-13: 10.24 sec ± 0.57 sec; Vehicle: 14.17 sec ± 0.76 sec; p<0.05; Fig 2A). Also 7 and 14 days after injury, the time taken the ND-13-treated animals to cross the bridge and reach the goal-box was significantly lower, compared to the control group. In the pole test, 2 days after injury, the group treated with ND-13 descended the pole faster than the group treated with saline (ND-13: 6.26 sec ± 0.13 sec; Vehicle: 7.41 sec ± 0.64 sec; p<0.05, Fig 2B). In the weeks following ischemia, the difference between the groups decreased, but the trend was still clear, as the animals treated with ND-13 performed better than those that received saline. Also in the cylinder test, ND-13 significantly attenuated motor asymmetry in the group treated, compared to the control group, 2 days after injury (ND-13: 0.10 ± 0.05; Vehicle: 0.30 sec ± 0.05; p<0.05, Fig 2C). The effect of ND-13 was still consistent 21 days after surgery: ND-13 treated mice used both paws more equally than mice in the control group.

### DJ-1 KO mice show higher sensitivity and less spontaneous recovery after focal ischemic injury compared to C57BL/6 wild type mice

**Fig 3:**
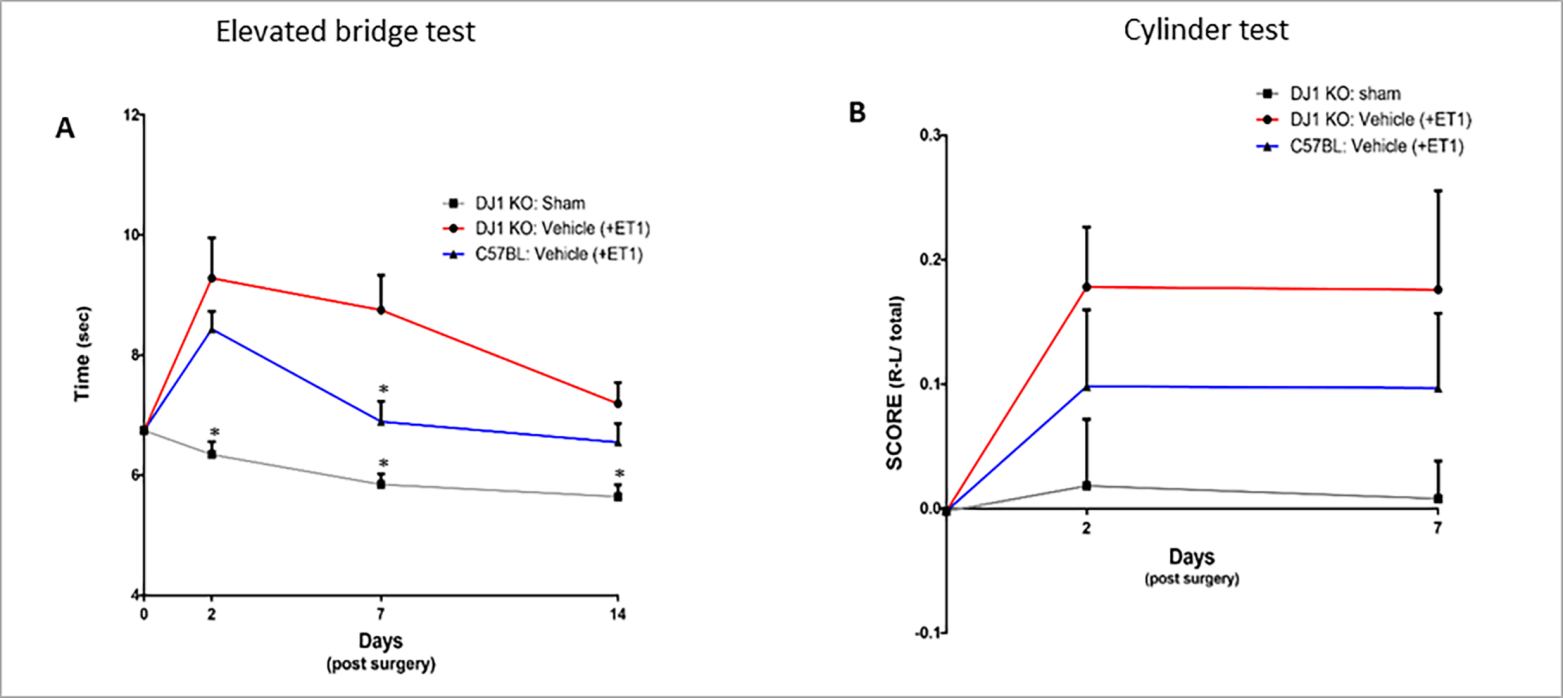
DJ-1 KO mice show higher sensitivity and less spontaneous recovery after ischemic injury compared to C57BL/6 mice. DJ-1 KO mice show slower spontaneous recovery compared to C57BL/6 mice and are more sensitive to ischemic insult. DJ-1 KO mice spent significantly more time crossing the beam in the elevated bridge test (A, p<0.05) than wild type mice. DJ-1 KO mice show only 5% recovery from day 2 to days 7 after injury, compared to C57BL/6 mice that improved by 20% over the same period of time. Improvement was also noted in cylinder test 7 days after injury (B). (Data is shown as mean ± SEM).

In order to evaluate the effect of DJ-1 deficiency on ischemic injury, the effect of ET-1 injection to DJ-1 KO mice was compared to that on C57BL/6 mice. Both groups received ET-1 injection into the right striatum to induce focal ischemic injury that resulted in motor dysfunction. DJ-1 KO mice showed higher sensitivity to ischemic damage and slower recovery compared to C57BL/6 mice. Motor function was reduced in the DJ-1 KO group by 21% as measured by the elevated bridge test, 7 days after injury (DJ-1 KO: 8.75 sec ± 0.57 sec; C57BL/6: 6.89 sec ± 0.34 sec; p<0.05; Fig 3A). Recovery was measured by the improvement in time taken to cross the bridge on measurement days. Recovery was slower in DJ-1 KO mice, with only 5% improvement from day 2 to day 7 after injury, compared to C57BL/6 mice that improved by 20% (Fig 3A). In the cylinder test, the effect was not statistically significant but the same trend was observed 2 days and 7 days after ischemic injury (Fig 3B).

### ND-13 improves motor function of DJ-1 KO in mouse model of focal ischemic injury

**Fig 4:**
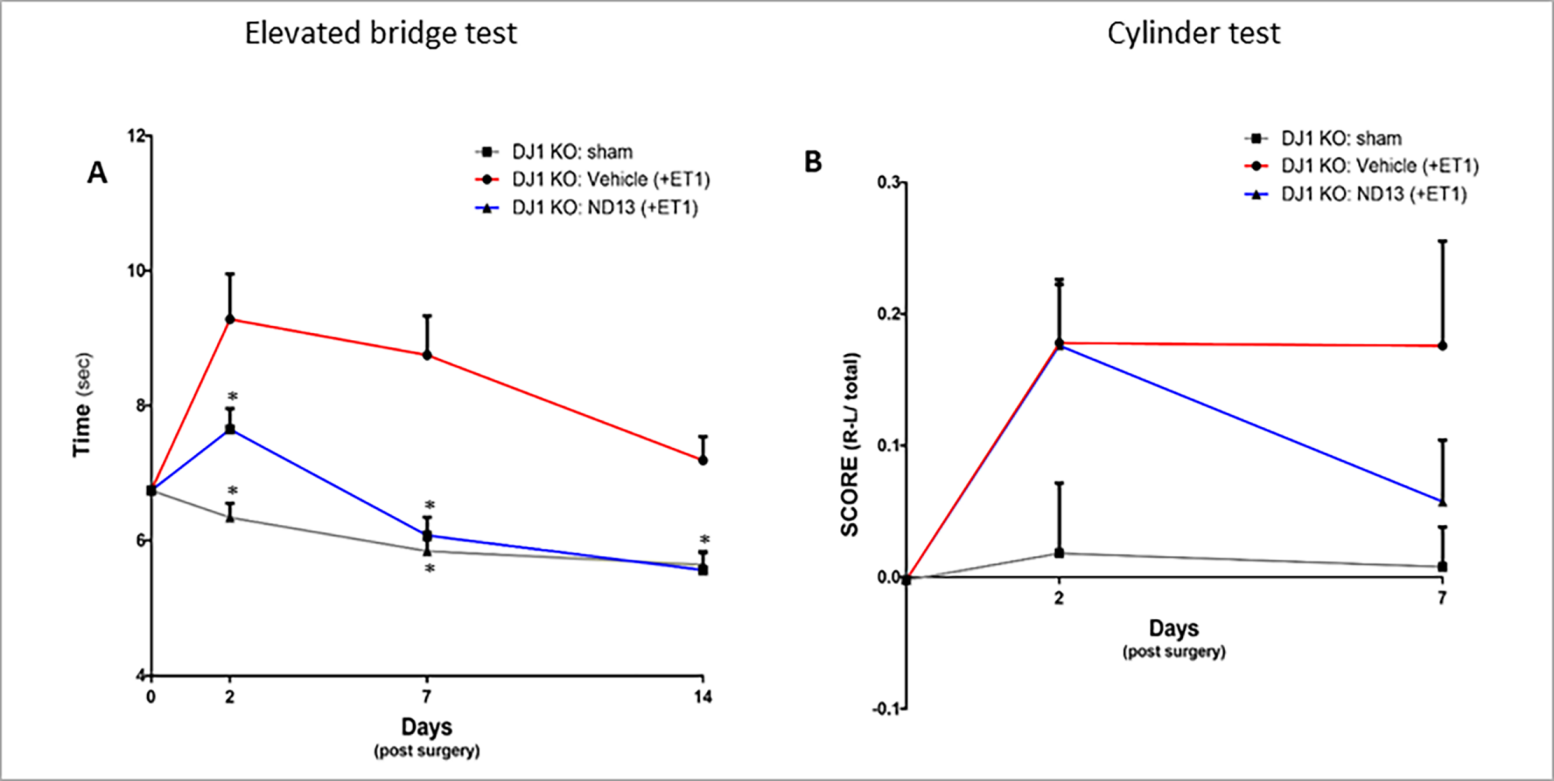
Effect of ND-13 treatment on functional recovery of DJ-1 KO mice after ET-1 induced focal ischemic injury. DJ-1 KO mice treated with ND-13 show improvement 2 days after ischemic injury in the elevated bridge test as they spend less time crossing the beam than wild type mice (A, p<0.05). This effect was consistent for at least two weeks following injury. Improvement was also noted in cylinder test 7 days after injury (B). (Data is shown as mean ± SEM).

To study the effect of ND-13 in the absence of DJ-1, we used the ET-1 model on DJ-1 KO mice. After ET-1 injection into the right striatum, DJ-1 KO mice received ND-13 or vehicle (saline) twice a day for 5 days. The ND-13-treated group showed significant improvement in motor behavior, even in the absence of endogenous DJ-1, compared to the control group. In the elevated bridge test, treatment with ND-13 decreased time to cross the beam by 30%, compared to vehicle treatment, 7 days after injury (ND-13: 6.08 sec ± 0.27 sec; Vehicle: 8.75 sec ± 0.57 sec; p<0.05; Fig 4A). The effect was still significant two weeks after the injury. In the cylinder test, DJ-1 KO mice treated with ND-13 showed a reduction in motor asymmetry in comparison to the control group, 7 days after injury (ND-13: 0.058 ± 0.04; Vehicle: 0.188 ± 0.08; Fig 4B). Results were not significant but a trend was noticed.

### Proteomics analysis reveals protein level changes following treatment of ischemic wild type mice with ND-13

Two experimental groups of ET-1 ischemic C57BL/6 mice, treated with ND-13 or saline, were tested for protein expression level changes (see methods). Average expression for each protein and fold changes (FC) between the tested groups were calculated. Changes were considered significant only if p<0.05 and the fold change>2. Out of all proteins that were found in the analysis, expression levels of 39 proteins changed following ND-13 treatment compared to vehicle treatment. Here we focus on the 7 of these proteins that have a link to the ischemic insult and may help explain some of the effects of the treatment.

**Fig 5:**
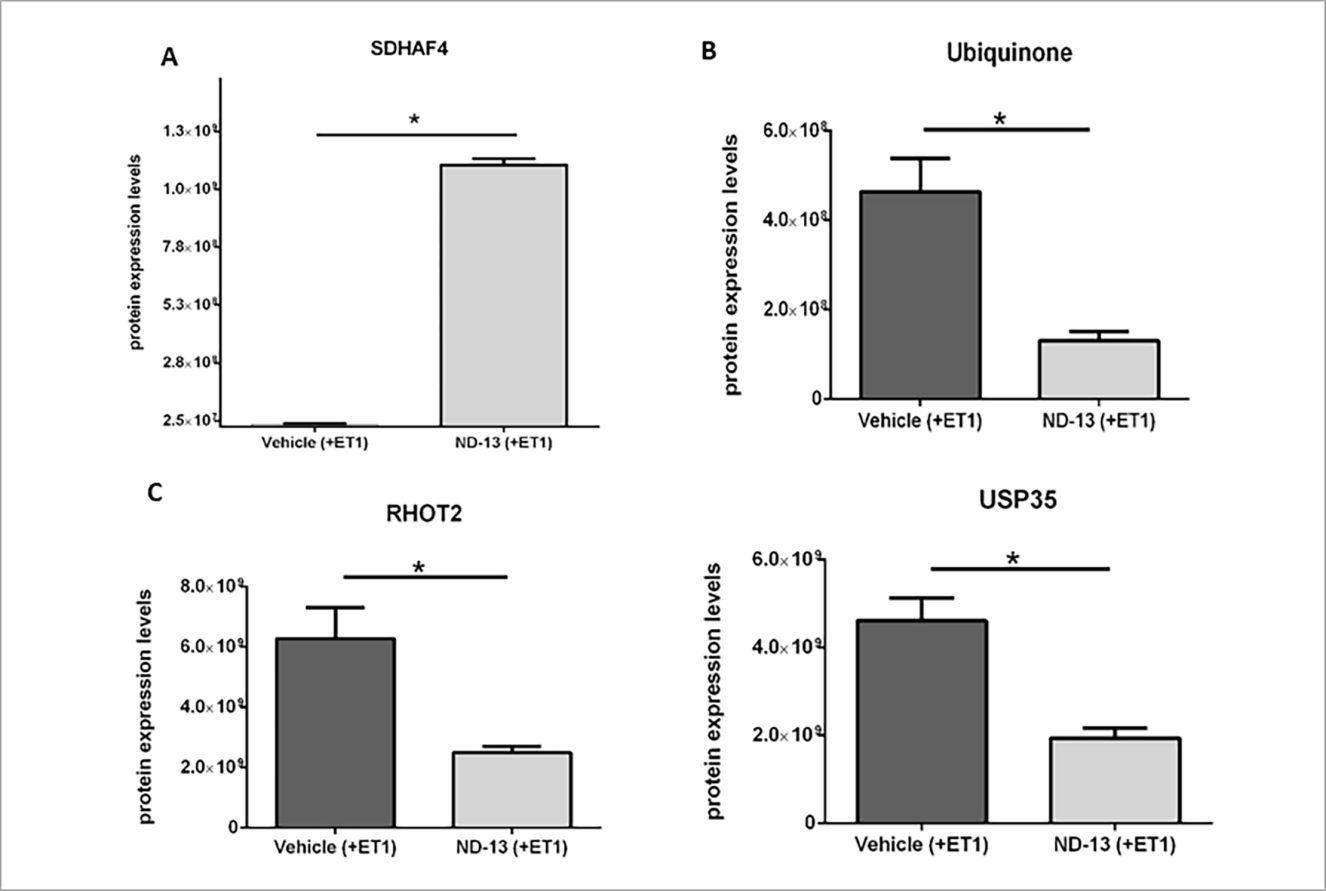
Changes in mitochondrial protein expression levels following ND-13 treatment. Ischemic wild type mice treated with ND-13 show significant change in protein expression levels compared to saline treated ischemic wild type mice. SDHAF4 protein levels increased after ND-13 treatment (A, FC = 107.4, p<0.001). Ubiquinone protein levels decreased after ND-13 treatment (B, FC = 3.5, p<0.01). Rhot2 protein levels decreased after ND-13 treatment (C, FC = 2.5, p<0.01). Usp35 protein levels decreased after ND-13 treatment (D, FC = 2.4, p<0.01). (Data is shown as mean ± SEM).

Previous reports show the effect of ND-13 on the mitochondria. Treatment with ND-13 helped preserve mitochondrial membrane potential, thus stabilizing mitochondrial function in the presence of 3NP toxin that inhibits succinate dehydrogenase activity [35]. Here, we have identified an increase in the levels of mitochondrial protein succinate dehydrogenase assembly factor 4 (SDHAF4) following ND-13 treatment (ND-13: 1.13E+09 ±2.51E+07; Vehicle: 1.05E+07 ±2.31E+06; fold change: 107.4, p<0.001; Fig 5A). SDHAF4 has a protective role on the mitochondria against oxidative stress: It enhances mitochondrial succinate dehydrogenase (SDH) activity, promoting the blocking of excess reactive oxygen species (ROS). Furthermore, SDHAF4 mutants display neuronal dysfunction, neurodegeneration, and sensitivity to oxidative stress [45–46]. Our data indicates a higher degree of mitochondrial preservation after ND-13 treatment.

SDH complex (also known as respiratory complex II), a part of the mitochondrial electron transport chain, elicits reduction of ubiquinone (coenzyme-Q) to ubiquinol, and therefore promotes energy production and antioxidant protection [47–49]. We found a reduction in the expression levels of ubiquinone following treatment with ND-13, corresponding to SDH-complex activity (ND-13: 1.30E+08 ±1.97E+07; Vehicle: 4.64E+08 ±7.51E+07; fold change: 3.5, p<0.01; Fig 5B).

Another key component of the oxidative stress response is the antioxidant enzyme SOD1 (copper-zinc superoxide dismutase) that provides defense against reactive oxygen species (ROS), scavenging superoxide radicals [50,51]. The activation of SOD1 is dependent on copper incorporation at the active site, a complex and highly regulated process [52,53]. The final step in SOD1 maturation is the formation of homodimers. Copper homeostasis protein COMMD1 (copper metabolism Murr1 domain containing 1) regulates the activation of SOD1. COMMD1 impairs SOD1 activity by reducing the expression levels of enzymatically active SOD1 homodimers late in the post-translational maturation process [54]. We found that after ND-13 treatment, COMMD1 protein levels decreased compared to vehicle treatment (ND-13: 7.45E+05; Vehicle: 8.01E+06; fold change: 10.76, p<0.05). This downregulation of COMMD1 suggests less impairment of SOD1 activity.

We also found that after treatment with ND-13, mitochondrial Rho GTPase 2 (RHOT2, Miro2) protein expression levels decreased compared to vehicle treatment (ND-13: 2.49E+09 ±2.16E+08; Vehicle: 6.28E+09 ±1.02E+09; fold change: 2.5, p<0.01; Fig 5C). As a Miro protein, RHOT2 is involved in mitochondrial homeostasis and apoptosis, indicating dysfunctional mitochondria elimination following ND-13 treatment. Furthermore, USP35 (Ubiquitin Specific Peptidase 35), a mitochondrial deubiquitinating enzyme, can delay parkin mediated mitophagy. Upon mitochondrial depolarization, USP35 dissociates from damaged mitochondria, allowing parkin activity. In the absence of USP35, the mitophagy increases [55]. Indeed, we found a decrease in USP35 protein expression levels following ND-13 treatment (ND-13: 1.94E+09 ±2.23E+08; Vehicle: 4.61E+09 ±5.19E+08; fold change: 2.4, p<0.01; Fig 5D) corresponding to the increase in mitophagy.

### Changes in proteins involved in potassium channel regulation following treatment with ND-13

**Fig 6:**
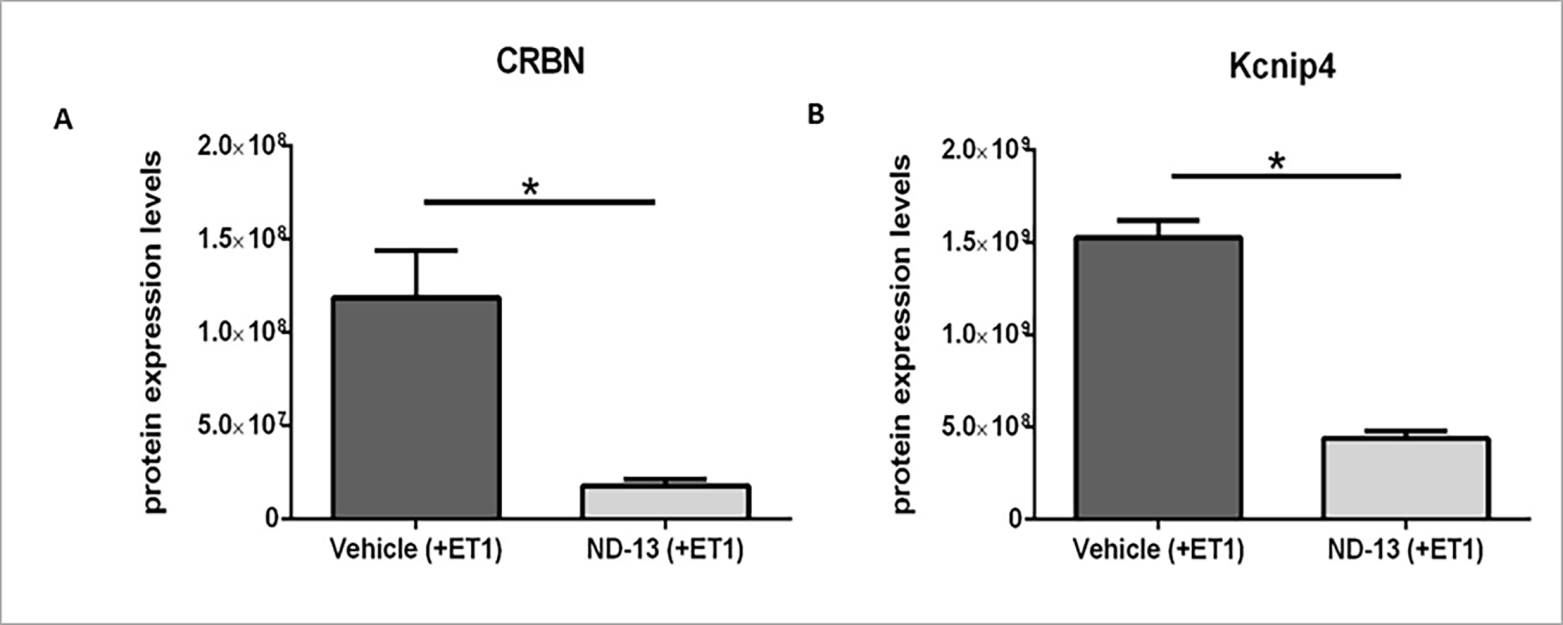
Changes in potassium channel regulators protein expression levels following ND-13 treatment. Ischemic WT mice treated with ND-13 show significant change in protein expression levels compared to WT mice treated with saline, 2 days after injury. CRBN protein levels decreased after ND-13 treatment (A, FC = 6.7, p<0.01). Kcnip4 protein levels decreased after ND-13 treatment (B, FC = 3.4, p<0.01). (Data is shown as mean ± SEM).

The opening of K+ channels mediates feedback control of excitability in a variety of conditions. Voltage gated K+ channels help bring the activated membrane more rapidly back toward its original negative potential [56]. The large-conductance voltage- and Ca2+-activated K+ channel (BK channel) improve the survival of neurons exposed to ischemic conditions, since their activation tends to reduce cellular excitability [57]. Cereblon protein (CRBN) interaction with the BK channel reduces the surface expression of functional channels. Therefore, CRBN plays a role in modulating neuronal BK channel activity [58]. We have identified a decrease in the levels of CRBN following ND-13 treatment (ND-13: 1.77E+07 ±3.55E+06; Vehicle: 1.19E+08 ±2.52E+07; fold change: 6.7, p<0.01; Fig 6A), suggesting an increase in the surface expression of functional KB channels that, in turn, improve the survival of cells after ischemia.

Another type of potassium channel that may be involved in reducing ischemic injury damage is the Kvα4 (Shal-related) voltage-gated rapidly inactivating A-type potassium channels. upregulation of A-type currents (IA) after ischemia correlates with higher resistance of cells to ischemic insult by decreasing excitotoxicity [59]. The protein Kcnip4 (Potassium Voltage-Gated Channel Interacting Protein 4) is a regulatory subunit of Kv4 channels that mediate the neuronal *IA* currents. Unlike other K-channel interacting proteins, Kcnip4 largely reduces surface expression of the Kv4 channel complexes [60]. We show that Kcnip4 levels decrease after ND-13 treatment (ND-13: 4.38E+08 ±4.19E+07; Vehicle: 1.52E+09 ±9.09E+07; fold change: 3.4, p<0.01; Fig 6B). This may suggest an increase in A-type channel expression and subsequent reduction in excitability and ischemic damage.

## Discussion

The present study shows the benefits of treatment with the novel DJ-1 based peptide, ND-13, on focal ischemic injury in mice. Using the vasoconstrictor Endothelin-1 (ET-1), we induced ischemic injury in separate groups of DJ-1 KO and C57BL/6 mice, which resulted in tissue damage and motor dysfunction [44]. To assess the effects of ND-13 treatment, motor function behavioral tests were performed. The motor capabilities of both DJ-1 KO and C57BL/6 groups significantly improved after subcutaneous administration of ND-13, following Ischemic injury. Previous studies show that treatment with ND-13 provides protection and promotes survival in mouse models of neurodegenerative diseases characterized by severe motor dysfunction, including Parkinson's disease and multiple system atrophy [34,35]. Our study expands these findings, demonstrating that treatment with ND-13 improves functional recovery from ischemic injury by promoting tissue survival.

Ischemic stroke is a common cause of permanent disability in adults worldwide. Surviving the initial injury usually leads to a long-term loss or limitations of function and the need for a long and agonizing rehabilitation [61,62]. Motor impairments are the most common results of stroke and affect an individual's ability to complete everyday activities and participate in everyday life situations [63,64]. Here we demonstrate a therapeutic effect of treatment with ND-13 in a mouse model of focal ischemic injury. ND-13 reduced motor dysfunction and increased recovery after injury. Significant reduction in motor asymmetry, improvement in body balance and motor coordination were observed after ND-13 treatment, compared to the control group.

Further experiments on DJ-1 Knock-out (KO) mice revealed that DJ-1 KO mice show higher sensitivity and less spontaneous recovery from striatal ET-1-induced ischemic injury, compared to C57BL/6 mice. These findings are consistent with the notion that DJ-1 participates in the endogenous neuroprotection after stroke. In various rodent models, it has been reported that loss of DJ-1 increases the sensitivity to excitotoxicity after ischemia, whereas elevated expression of DJ-1 can reverse this sensitivity and provide further protection through alleviation of oxidative stress [30,65]. DJ-1 is detected immediately after stroke and efficiently translocated into the mitochondria and may contribute to mitochondria-mediated neuroprotection [19]. Furthermore, oxidative stress induces the release of DJ-1 in reactive astrocytes, scavenges free radicals and reduces cell injury [66]. We show that ND-13 provides compensation for DJ-1 deficits in DJ-1 KO mice. This suggests that ND-13 works in a DJ-1 independent manner. That is, the presence of the endogenous DJ-1 is not required for ND-13 activity, and even in the absence of DJ-1, ND-13 improves motor function and recovery significantly after ischemic injury.

It is well known that stroke leads to increased production of free radicals and reactive oxygen species (ROS) in the brain [67,68], and to accumulation of glutamate and excessive activation of glutamate receptors [4,69]. Both responses, which eventually lead to cell vulnerability and neuronal death, are sequential but also interacting processes and the close relationship between these responses is well defined [4,12,70,71]. Our objective of demonstrating a basis for clinical application of the ND-13 peptide to enhance neuroprotection in stroke patients led us to choose C57BL/6 mice (as opposed to DJ-1 KO mice) for the proteomic analysis. To the extent that mice data can be translated to humans, clearly wild type mice are more relevant than gene “knock out” models.

In this study, we show an approach to minimize neuronal damage and improve functional recovery through the simultaneous regulation of different pathways.

A detailed proteomic analysis of protein expression levels after ND-13 treatment to ischemic C57BL/6 mice revealed significant changes in several regulatory proteins involved in oxidative stress and neurotoxicity responses.

Changes in the expression levels of several proteins involved in regulation of mitochondrial function were observed in response to ND-13 administration. These changes can help promote the anti-oxidative stress response, preserve mitochondrial function and regulate the elimination of damaged mitochondria, which encourages cell survival. Other proteins found in the analysis regulate various potassium channels. Voltage gated potassium channels are major mediators of excitability in the brain, and help reduce membrane potential [72]. The observed downregulation of these proteins after ND-13 treatment, can lead to reduction in excitability of the tissue after stroke and consequently lessen tissue damage, thereby leading to improved function.

In spite of the above, there is still not enough evidence on the mechanism of action of ND-13 and further research will have to be undertaken. The present study provides a proof of concept, justifying further evaluation of treatment with ND-13 for stroke. The advantages of the ET-1 focal ischemic model are its simplicity, reliability and the option to choose the damage site. A further study using other models, such as the middle cerebral artery occlusion model, is needed to better understand the clinical relevance of these results. Also, in order to implement our findings in the clinic, further research is needed to measure neurological and histological parameters and determine the safety of ND-13, its stability, optimal mode of administration and effective doses.

In conclusion, our findings propose a new therapeutic target for ischemic stroke. Treatment with ND-13 enhances functional recovery and may play a significant role in neuroprotection after ischemic injury. These findings have important implications and could benefit patients with ischemic stroke.
